# Hypovolemia with peripheral edema: What is wrong?

**DOI:** 10.1186/s13054-023-04496-5

**Published:** 2023-05-27

**Authors:** Randal O. Dull, Robert G. Hahn

**Affiliations:** 1 Department of Anesthesiology, University of Arizona College of Medicine, 1501 N. Campbell Avenue, Suite 4401, PO Box 245114, Tucson, AZ 85724-5114 USA; 2 Department of Pathology, University of Arizona College of Medicine, Tucson, AZ USA; 3 Department of Surgery, University of Arizona College of Medicine, Tucson, AZ USA; 4 Karolinska Institute at Danderyds Hospital (KIDS), 171 77 Stockholm, Sweden

## Abstract

Fluid normally exchanges freely between the plasma and interstitial space and is returned primarily via the lymphatic system. This balance can be disturbed by diseases and medications. In inflammatory disease states, such as sepsis, the return flow of fluid from the interstitial space to the plasma seems to be very slow, which promotes the well-known triad of hypovolemia, hypoalbuminemia, and peripheral edema. Similarly, general anesthesia, for example, even without mechanical ventilation, increases accumulation of infused crystalloid fluid in a slowly equilibrating fraction of the extravascular compartment. Herein, we have combined data from fluid kinetic trials with previously unconnected mechanisms of inflammation, interstitial fluid physiology and lymphatic pathology to synthesize a novel explanation for common and clinically relevant examples of circulatory dysregulation. Experimental studies suggest that two key mechanisms contribute to the combination of hypovolemia, hypoalbuminemia and edema; (1) acute lowering of the interstitial pressure by inflammatory mediators such as TNFα, IL-1β, and IL-6 and, (2) nitric oxide-induced inhibition of intrinsic lymphatic pumping.

## Introduction

The clinician is frequently confronted with the combination of hypovolemia and peripheral edema. In healthy humans, compensatory mechanisms including lymphatic return of extravascular fluid and transcapillary Starling forces act in concert to attenuate edema by increasing plasma volume. When this does not occur, treatment is a challenge, as administration of a diuretic will likely worsen hypovolemia and cause hypotension, while fluid administration worsens the edema. The most common explanation of this confusing condition is that capillary permeability has increased due to damage to the endothelial glycocalyx layer. However, increased capillary leakage normally accelerates lymphatic flow which normalizes the balance between the body fluid volumes, and might even increase the plasma albumin concentration (“interstitial washdown”) [1, 2].

This paper discusses how inflammatory insults and certain drugs may disturb the normal exchange of infused crystalloid fluid between the plasma and the interstitial space by mechanisms other than increased capillary leakage. Our review focuses on the possibility that decreased lymphatic return of fluid and protein alone can create hypovolemia, hypoalbuminemia, and peripheral edema. The pathophysiology responsible for poor lymphatic return might involve a reduction of the interstitial hydrostatic pressure (P_i_) or failure of the intrinsic contractility of lymphatic smooth muscle cells. These mechanisms are distinctly different from lymphatic obstruction due to tumors, compression during compartment syndromes and resistance to thoracic duct outflow.

## Volume kinetic simulation

**Fig. 1:**
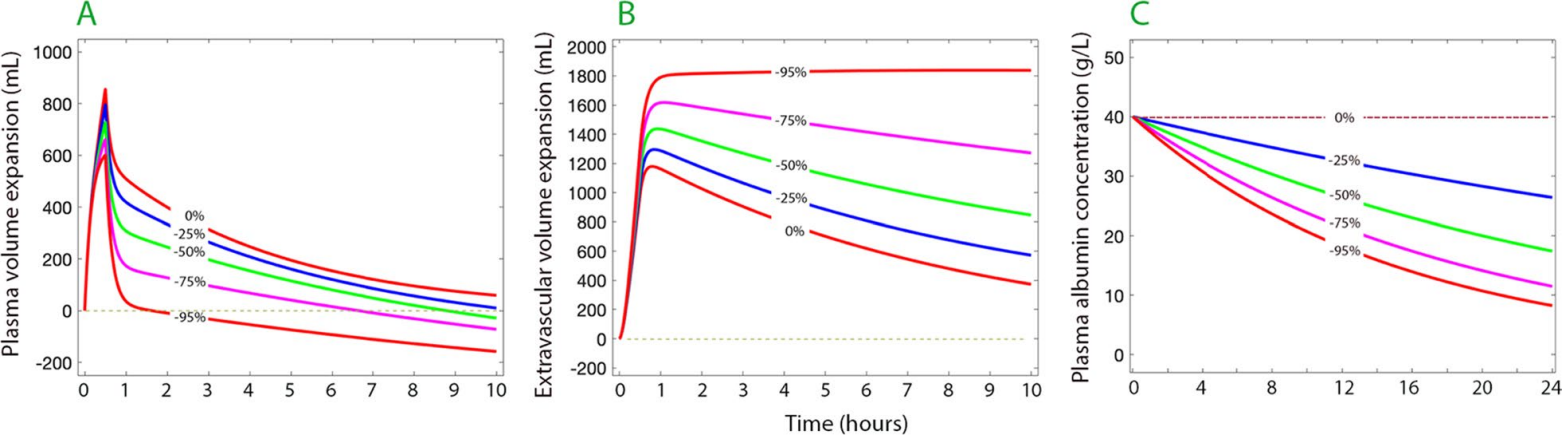
Computer simulated volume expansion of the plasma (**A**), extravascular space (**B**) and the plasma albumin concentration (**C**) in response to infusion of 30 mL/kg over 30 min followed by 15 mL/kg over 1 h of Ringer´s acetate/lactate. The percentages imply the degree of inflicted restriction of lymphatic return of fluid and albumin. The albumin kinetics assumes a capillary leakage of 5% of the intravascular pool per hour which, at steady state, is fully returned by lymphatic flow, and is unaffected by fluid loading. The volume kinetic parameters were taken from a mixed population of conscious and anesthetized patients [4]. Each gram of albumin was modeled to bind 10 mL of fluid [5]. The extravascular volume expansion could not be divided into a fast and slowly exchanging pool because it is unknown from which pool the lymph originates

Volume kinetics is a macroscopic method that is useful for the study of whole-body fluid maldistribution [3]. Simulations based on volume kinetic theory confirm that inhibition of lymphatic flow is likely to create marked disturbances of fluid and albumin distribution even when capillary filtration is normal and no other pathophysiological disturbance is present (Fig. 1). This model and our synthesis of evidence explaining the combination of hypovolemia and peripheral edema is focused on distributive changes between the plasma and the interstitial space (and, importantly, sub-compartments therein). This analysis contrasts with the macroscopic changes between the stressed and unstressed plasma volumes that are part of hemodynamic derangements which occur solely within the vascular compartment.

## Third fluid space

Volume kinetic studies performed in sheep 20 years ago demonstrated that the normal balance between plasma and interstitial fluid volumes is disturbed by isoflurane anesthesia. Brauer et al*.* [6] found that the disappearance of infused 0.9% saline from the kinetic system far exceeded the urine flow and was assumed to have entered an undefined “third space”. A follow-up study showed that isoflurane and not mechanical ventilation was the cause of this unaccounted fluid [7]. A subsequent study of humans undergoing thyroid surgery reported that isoflurane and propofol anesthesia to a similar degree excluded substantial amounts of distributed fluid from being reabsorbed to the plasma [8]. At this time, it became apparent that the interstitium contains two kinetic fluid pools, one that exchanges rapidly and another that equilibrates more slowly with the plasma, the latter being difficult to quantify by means other than population kinetic analysis.

## Inhibited urine output

Anesthesia also reduces the urinary output. Norberg et al*.* demonstrated that isoflurane reduces the rate constant (*k*_10_) for urinary excretion by 50% in anesthetized but spontaneously breathing volunteers [9]. A study of conscious and anesthetized mechanically ventilated humans ascribed the reduction of *k*_10_ to low arterial pressure [4]. Interestingly, the anesthesia-induced reduction of the urinary excretion is inversely correlated with the accumulation of fluid in the slowly equilibrating interstitial fluid pool [10].

## Interstitial space: structure–function

Infused crystalloid fluid distributes between the plasma volume (3 L) and expandable parts of the interstitial space with a half-time of approximately 25–30 min. Volume kinetics identifies two fluid compartments in the interstitial space that equilibrate with the plasma but at different rates. The rapidly exchanging pool has a size similar to the plasma volume. Yet unpublished data from our group suggest that the expandable volume of the slowly exchanging pool is of negligible size when a few hundred mL are infused, while infusion of larger volumes of Ringer´s solution expand a progressively larger volume. During inflammatory states, the slowly exchanging pool expands even more and becomes larger than physiologically possible, which suggests that fluid can be bound or sequestered within the slow pool. Does this macroscopic view agree with anatomical and physiological studies of the interstitial fluid space?

**Fig. 2:**
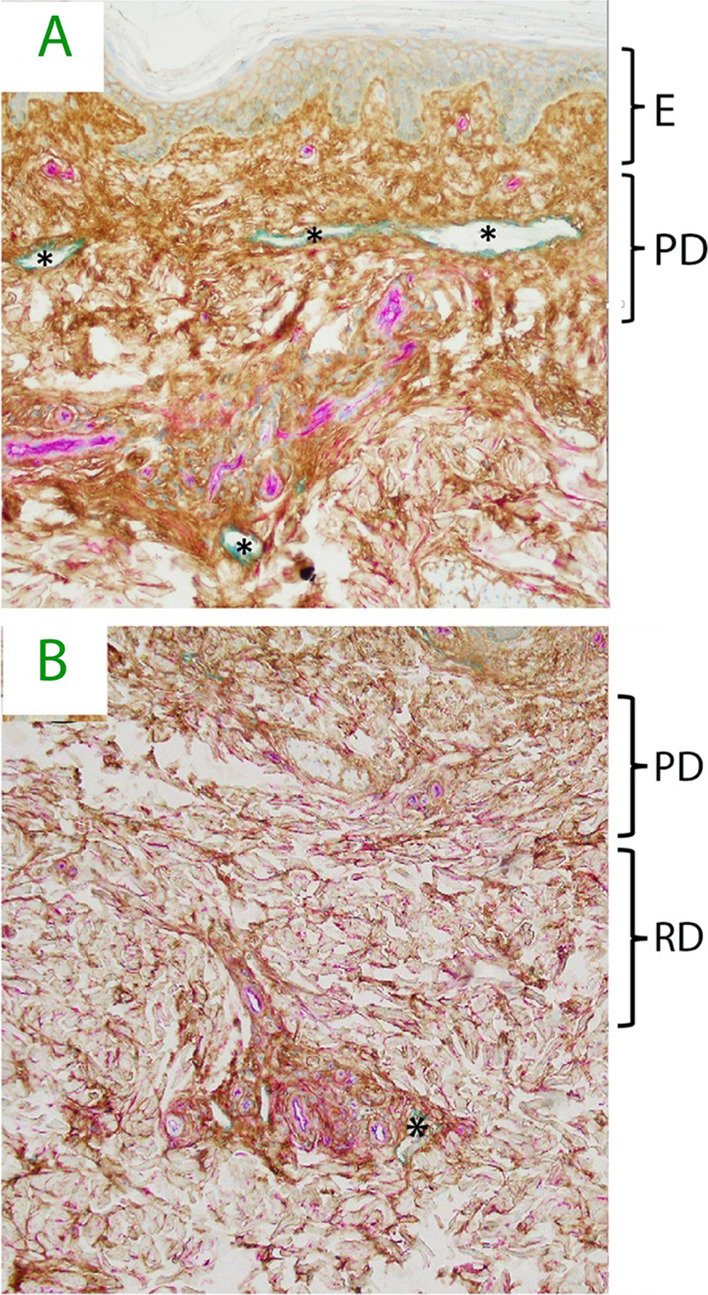
**A** Human dermis histology demonstrating hyaluronan staining (HA, brown), vascular endothelial cell of capillaries, small arteries and veins (CD34, magenta) and lymphatic vessels (LYVE-1, teal). Note intercellular HA in epidermis and abundant HA filling interstitial space in papillary dermis. Lymphatic vessels (_*_) are common in papillary dermis. **B** Reticular dermis. Dense HA staining is seen around peri-vascular bundles and filling inter-cellular space. Lymphatics (_*_) are much less common in reticular dermis. E = epidermis, PD = papillary dermis, RD = reticular dermis. Courtesy by Dr. Neil Theise and coworkers, experiments are explained in references 13 and 14

Recent advances in structural characterization of the interstitial space have revealed much complexity. Hyaluronan (HA) fills much of the interstitial space and forms a gel phase [11, 12]. A recent finding is an extensive continuity of the interstitial space across tissue and organ boundaries that allows for the long-distance movement of free fluid [13, 14]. Lymphatic vessels serve to clear the interstitial space from fluid and proteins, which keeps the interstitial space relatively dry and, therefore, determines interstitial fluid pressure (P_if_, Fig. 2). The lymphatic system originates as primary lymphatic vessels, which are highly permeable and allow interstitial fluid and proteins easy access into the lymphatic system. Primary lymphatics coalesce into larger secondary lymphatic vessels and then into collecting lymphatics, the later which posses’ circumferential lymphatic smooth muscle cells that impart contractile forces and endothelial-lined valves that limit uni-direction fluid flow [15]. Lymphatic vessels are typically found near the arteriole-venous bundle.

Fluid in the interstitial space consists both of free fluid and fluid associated with the gel phase [16]. The fibers and bundles of collagen and other filamentous matrix proteins create a lattice that excludes most serum proteins creating the “excluded volume”, e.g., the total fluid volume where serum proteins cannot harbor. This limits serum proteins to a small volume of free fluid outside the gel phase [17, 18] and in some areas colloid osmotic pressure might be higher than expected.

Surface charges on macromolecules attract electrolytes that contribute to the net osmotic force exerted by proteins and proteoglycans, a process called the Gibbs-Donnan equilibrium [19]. The Donnan osmotic pressure contributes by approximately one third to one-half of the gel-related influence on P_if_ [20]. However, most clinically relevant crystalloid infusion fluids differ only slightly in their electrolyte composition which have little, if any, effect on fluid kinetics [21] and, therefore, no demonstrable effects on interstitial fluid pressure. The role of extracellular proteoglycans on electrolyte buffering remains an unsettled hypothesis [22].

## Fluid distribution during general anesthesia

**Fig. 3:**
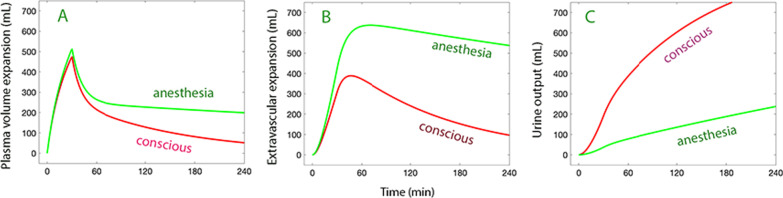
Distribution of an intravenous infusion of 15 ml/kg of buffered Ringer´s solution over 30 min followed by 10 mL/kg over 60 min between (**A**) the plasma (**B**) extravascular space, and (**C**) urine in a human weighing 65 kg (total volume 1620 mL) depending on whether he/she is conscious or under general anesthesia. Simulation based on volume kinetic parameters from 157 conscious volunteers and 85 anesthetized patients [10]. The extravascular expansion is the sum of the rapidly and the slowly equilibrating pool of fluid

General anesthesia creates a moderately severe maldistribution of fluid that is dominated by the strong inhibition of the diuretic response to volume expansion [6, 10]. The anesthesia-induced decrease of arterial pressure unloads baroreceptors which, in turn, activates renal sympathetic nerves [23]. These nerves increase sodium and water reabsorption and cause renal vasoconstriction, all leading to reduced urine output (Fig. 3).

Another finding during general anesthesia is that rate constant for fluid distribution to the slowly equilibrating interstitial pool is increased [10]. The precise mechanism is not known, but one possibility is that the low urine output maintains an expanded central (plasma) volume and therefore a higher capillary hydrostatic pressure (P_c_) that forces fluid into the slow exchange pool. This is supported by the observations that small amounts of crystalloid fluid (300 mL during 15 min) expand mainly the plasma volume [24] while larger infused volumes, combined with restricted urine flow, promotes the development of a bi-exponential plasma dilution curve, indicating the slowly developing and prominent extravascular distribution that is illustrated in Fig. 3. By contrast, moderate volume loading associated with a prompt diuretic response creates a mono-exponential curve where the single fluid space attains a size being twice the plasma volume [25] which may represent the sum of the plasma and the rapidly equilibrating fluid pool.

A complementary hypothesis is that general anesthesia initiates a mild form of the interstitial molecular events that occur during inflammation. These events are detailed below but include sequestration of fluid in the gel phase, resulting from a reduction of the interstitial hydrostatic pressure (P_if_), and inhibition of lymphatic pumping. A recent finding that indirectly supports this hypothesis is that poor return flow from the interstitium to the plasma remains for several hours after awakening from general anesthesia, while the sympathetic-induced inhibition of the diuretic response to volume expansion resolves almost instantly [26].

## Fluid movements in the lymphatic system

Movement of free interstitial fluid into the lymphatic system is promoted by two types of tissue forces: *extrinsic* and *intrinsic* forces [27]. Extrinsic forces result from tissue movement, for example, skeletal muscle contraction, breathing, peristalsis, arterial pulsations, and external massage. All these actions increase tissue pressure. The primary lymphatic vessels are capillary-like structures whose walls are made of loosely apposed endothelial cells that open in response to increased tissue pressure and allows fluid to enter the lymphatic capillaries. Compression garments aids in pushing fluid into the primary lymphatic vessels by increasing the tissue pressure.

Intrinsic forces refer to contractility of lymphatic smooth muscle cells. Lymphatic smooth muscle cells have characteristics of both smooth muscle and cardiac muscle cells which demonstrate spontaneous contractions and contractile forces that are modulated by preload, afterload, and contractility. In concert with uni-directional valves, intrinsic lymphatic pumping (rate and contractility) determines lymph flow rates.

## Poor return flow in severe disease

There are clinical conditions in which volume kinetic studies show that the return flow of fluid from the interstitium to the plasma (probably via the lymphatics) is severely reduced. These are the "transurethral resection syndrome" (studied in pigs) [28], sepsis (sheep) [29], and preeclampsia (humans) [3]. These three conditions are all characterized by hypovolemia, hypoalbuminemia, and peripheral edema**.** There is much evidence that inflammatory molecules play an important role in the maldistribution of fluid during acute inflammation, such as sepsis and burn injury, by reducing P_if_. To discuss these findings in detail we first give a brief review of the factors that determine P_if_.

## Interstitial fluid pressure (P_if_)

Prior to the mid-1960’s, P_if_ was assumed to be near zero or slightly positive relative to the atmosphere. Guyton and colleagues tested a wide range of experimental conditions and consistently measured a negative P_if_ in the range of − 3 to 7 mm Hg [30, 31] which more recently has been reported to be − 2 to − 3 mm Hg [32, 33]. The negative P_if_ is maintained by an imbibition (suction) pressure created by colloids in the gel phase [20, 30, 31]. Moreover, the interstitial space is under tensile stress created by fibroblast-mediated, integrin-dependent processes [34].

The mechanisms cited above keep the interstitial space dry and maintains a very low volume of free fluid, which has been suggested to occupy as little as 1% of the interstitial volume. The remaining interstitial fluid is bound in the gel phase [35]. By contrast, volume kinetic analyses suggest that the free fluid phase, which may also include the lymphatic volume, whole or in part, normally amounts to approximately the same volume as the plasma. However, it might occupy a smaller space in inflammatory conditions as more fluid becomes sequestrated in the slowly exchanging pool.

## Mechanisms creating negative P_if_

Analysis of interstitial pressure and volume during lymphatic failure draws data primarily from studies of the skin, as it is among the largest organ system of the body and is estimated to hold approximately 3 L of fluid, second only to skeletal muscle that accounts for approximately 20 L [36]. Peripheral edema is usually observed earliest in the skin and the interstitial space of the dermis and sub-dermis have been well characterized in terms of histology and physiology.

Injecting lipopolysaccharide or inflammatory molecules intravenously and, in some cases, directly into the skin, reduces P_if_ from −2 mmHg to between −7.5 and −10 mmHg within minutes [32, 34]. The most dramatic decrease in P_if_ occurs in burn injury, where values of 40 mmHg and greater have been reported [37–41]. Such pressure changes suck intravascular fluid into the interstitial space and further reduces lymphatic flow. How can this occur?

**Fig. 4:**
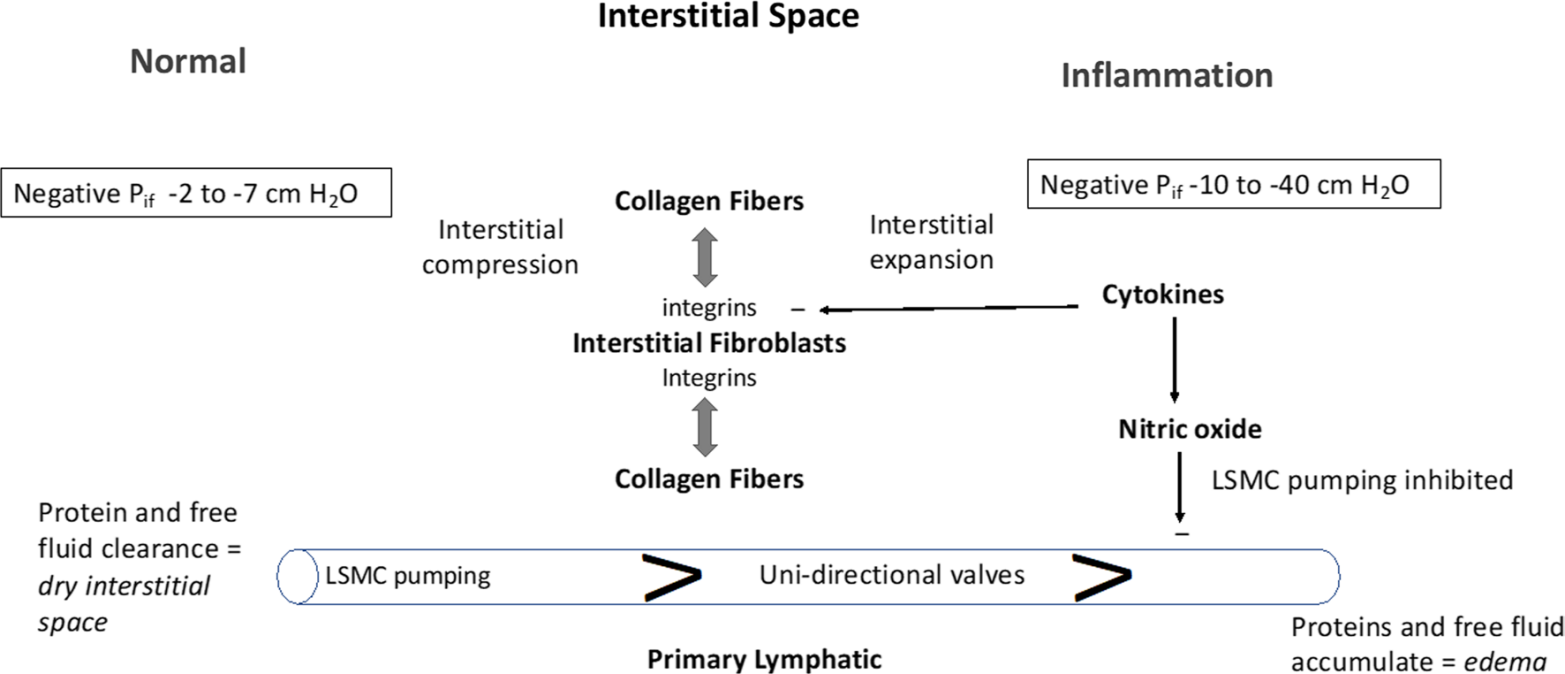
The interstitial space is represented in its Normal state (left side) and during Inflammation (right side). The interstitial space is normally contracted by integrin-dependent binding between interstitial fibroblasts and collagen fibers (tension indicated by double-headed arrows) that induces Interstitial Compression. In the Normal state, the interstitial space is kept “dry” by the action of lymphatic clearance and baseline pressure (P_if_) is slightly negative in the range of −2 to −7 cm H_2_O. Lymphatic clearance is dependent on activity of lymphatic smooth muscle cell pumping and functional uni-directional valves. During Inflammation, cytokines induce fibroblast-dependent relaxation and disassembly of the integrin-collagen complex, that promotes Interstitial Expansion and a more negative pressure (-10 to −40 cm H_2_O). Nitric oxide produced during inflammation has direct inhibitory actions on lymphatic smooth muscle cells to reduce the rate and force of lymphatic smooth muscle contraction; the reduced lymphatic clearance of fluid and proteins results in edema (bottom right)

The interstitial space is under tensile forces mediated by cell-collagen interactions and, specifically, by integrins. The basal tensile force sets the pressure–volume relationship. When tissue is excised and placed in isotonic saline it expands due to loss of these tensile forces [41, 42]. In a series of papers, Reed et al*.* have shown that inflammatory mediators reduce P_if_ to more negative values. Numerous cytokines reverse integrin-collagen binding, including histamine (mast cell degranulation and Compound 48/80), PAF, TNFα, IL-1β, IL-6, LPS, and IFN-γ [34] (Fig. 4).

This mechanism has also been studied in vitro using fibroblasts mixed into collagen gel. Fibroblasts bind to collagen via cell surface integrins that serve as protein linkages between the cell’s cytoskeleton and the collagen. The integrins are composed of heterodimers of α- and β-subunits, with both subunits having distinct members. There are four collagen-binding integrins: α_1_ β_1_, α_2_ β_2_, α_10_ β_1_, α_11_ β_1_ [43–46]. Fibroblasts generate tensile forces via integrin-collagen binding which contracts the interstitial space. Integrin-neutralizing monoclonal antibodies reduced P_if_ when injected intradermally, thus demonstrating the role of integrins in the P_if_-lowering process [47].

During acute inflammation, there is a biphasic change in P_if_. First, P_if_ becomes more negative due to the loss of tensile forces by cytokine-dependent interstitial relaxation. This expansion occurs rapidly and the ensuing more negative P_if_ increases fluid filtration and imbibes fluid from the capillary into the interstitial space. P_if_ starts at slightly negative pressures (− 2 to − 7 cm H_2_O) and might become deeply negative (− 10 to − 40 cm H_2_O). The shift can cause life-threatening hypovolemia during the first 6–8 h after burn injury [48] but negative changes in P_if_ are likely to be initially induced, to various degrees, in all conditions associated with acute inflammation. The presence of HA and glycosaminoglycans in plasma suggest that interstitial expansion is also associated with matrix fragmentation [49]. The second phase of this process—dominated by interstitial volume expansion shifts P_if_ towards zero, where the compliance characteristics for interstitial volume expansion cease [33].

## Lymphatic failure

The interstitial edema and hypovolemia become aggravated if lymphatic pumping is impaired. During sepsis and states of sustained inflammation, increased levels of nitric oxide (NO), likely generated by inducible nitric oxide synthase (iNOS), inhibits lymphatic smooth muscle cell pumping and, thus, contributes to lymphatic failure [50–52]. Slow return of albumin-rich lymph is the likely cause of maldistribution of albumin during inflammatory conditions, as the synthetic rate of albumin is normal during major abdominal surgery [53] and sepsis [54]. Moreover, laboratory experiments show that anesthesia drugs inhibit lymphatic pumping [55].

The combined effects of low P_if_ and poor lymphatic pumping greatly expands the interstitial space. As the interstitium fills with fluid, P_if_ shifts along its pressure–volume curve towards more positive values and eventually approaching zero, thus diminishing the forces for further fluid influx. During experimental thermal injury, the transition from largely negative P_if_ towards zero occurred over a time period of hours [40]. These changes allow fluid to enter the interstitial space with virtually no resistance, which causes pathological alterations in the interstitial architecture due to excessive amount of free fluid, e.g., ¨pitting¨ edema [35]. Lacune filled with fluid appears between the cells that may even lead to cellular hypoxia. The cytoskeleton of encapsulated organs might become disrupted, the severity of which varies slightly depending on the type of infused fluid [56].

Reduction of P_if_ increases the capillary leakage of blood plasma by a suction effect that does not necessarily involve alteration of the capillary permeability. This was illustrated by Arturson and Mellander in 1964 who studied the effects of second-grade burn injury on the cat paw; they found accelerated loss of intravascular fluid to the injured area but no change in the capillary filtration coefficient, which is a measure of capillary permeability [57].

## Capillary leakage syndromes

Hypovolemia, hypoalbuminemia, and peripheral edema occurs in several medical diseases and is generally attributed to increased capillary permeability, as reviewed by Siddall et al*.* [58]. Sepsis is the “type model” but others include snakebites, viral hemorrhagic fever, engraftment syndrome, and autoimmune diseases. Excessive capillary leakage may also be induced by cancer therapies based on drugs or immunoglobulins. These conditions are typically accompanied by increased cytokine levels, and administration of IL2, IL11, and IL12 as therapeutic efforts in cancer therapy has even induced capillary leakage syndromes.

A special case is the rare Idiopathic Capillary Leak Syndrome (Clarksons´disease) which presents dramatically with attacks of hypovolemic shock and pitting edema [58, 59]. Clarkson´s disease is not associated with inflammation [59] but acute serum from patients with Clarksons´s disease increases capillary leakage in experimental systems [60]. Polyvalent immunoglobulin is effective treatment, but endothelial hyperpermeability can also been counteracted by increasing intracellular cAMP using terbutaline (a beta_2_-receptor agonist) and theophylline [59, 60].

## Interstitial pressure in body organs

Much of our analysis has drawn upon data from the skin where P_if_ and the pressure–volume relationship is fairly accessible to interventions [61]. The situation is different in encapsulated organs. The *kidney* has an extensive network of specialized lymphatic vessels that are named for their intrarenal location [62]. Due to the renal capsule, P_if_ rises quickly as a result of external capsular compression, lymphatic obstruction (metastasis), systemic venous hypertension (e.g., congestive heart failure), ureteral obstruction, and increased capillary permeability. Parenchymal edema has been suggested to collapse the capsular and hilar lymph vessels and prevents lymph outflow, contributing to further pathological increases in P_if_ [63]. Renal interstitial edema and increased P_if_, is a typical finding in acute kidney injury (AKI) and is commonly associated with elements of intra-renal lymphatic failure [64, 65] likely due to compressive obstruction.

Sepsis is a leading cause of acute kidney injury [66] where the pathophysiology includes inflammatory-derived cytotoxic mediators, hemodynamic derangements, immune-cell infiltration. and lymphatic failure [67]. Sepsis-induced alterations share nitric oxide and its reactive oxidative metabolites as major pathological contributors [68, 69].

*Skeletal muscle* represents another encased organ system that develops compartment syndrome albeit more commonly due to cellular swelling rather than lymphatic failure. Skeletal muscles are composed of fibers arranged in bundles with each bundle surrounded by a connective tissue sheath [70]. Their lymphatic vessels differ from other lymph vessels in that they lack lymphatic smooth muscle cells [71]. Therefore, the pressure gradients for skeletal muscle lymph flow are generated solely by extrinsic forces created by muscle contraction. Owing to the fibrous sheath, any cellular swelling or increased capillary permeability and increase interstitial fluid pressure, results in a rapid rise in P_if_. The lymphatics have no ability to respond to increases in interstitial fluid as they too are encased.

The *gut* can develop both edema and compartment syndrome. The walls of the intestine are lined with an extensive population of leukocytes that, during acute inflammation, generate high levels of nitric oxide and cytokines [72]. In general, abdominal compartment syndrome results from mechanical factors caused by progressive tissue edema that, in part, includes lymphatic failure.

The mesenteric lymphatic vessels drain into the thoracic lymph duct and subsequently into the left subclavian vein. Increases in central venous pressure will reduce the pressure gradient for lymph flow and may worsen peripheral edema. Conditions commonly associated with elevated central venous pressure include heart failure, portal hypertension, volume overload, positive pressure ventilation, and positive end-expiratory pressure (PEEP). However, the outflow pressure which causes lymphangion stroke volume to begin to decrease is approximately 10 cm H_2_O [73] above which can cause lymphatic hypertension. Sustained increases in lymph vessel transmural pressure can result in adaptive responses that improve pumping efficiency mainly through increase in contractile rate [74]. However, lymphatic vessels cannot easily overcome large changes in outflow resistance [75]. Sustained mesenteric venous hypertension initially increases lymph flow but, over the course of days, attenuates lymphatic pumping [76].

## Macroscopic studies

**Fig. 5:**
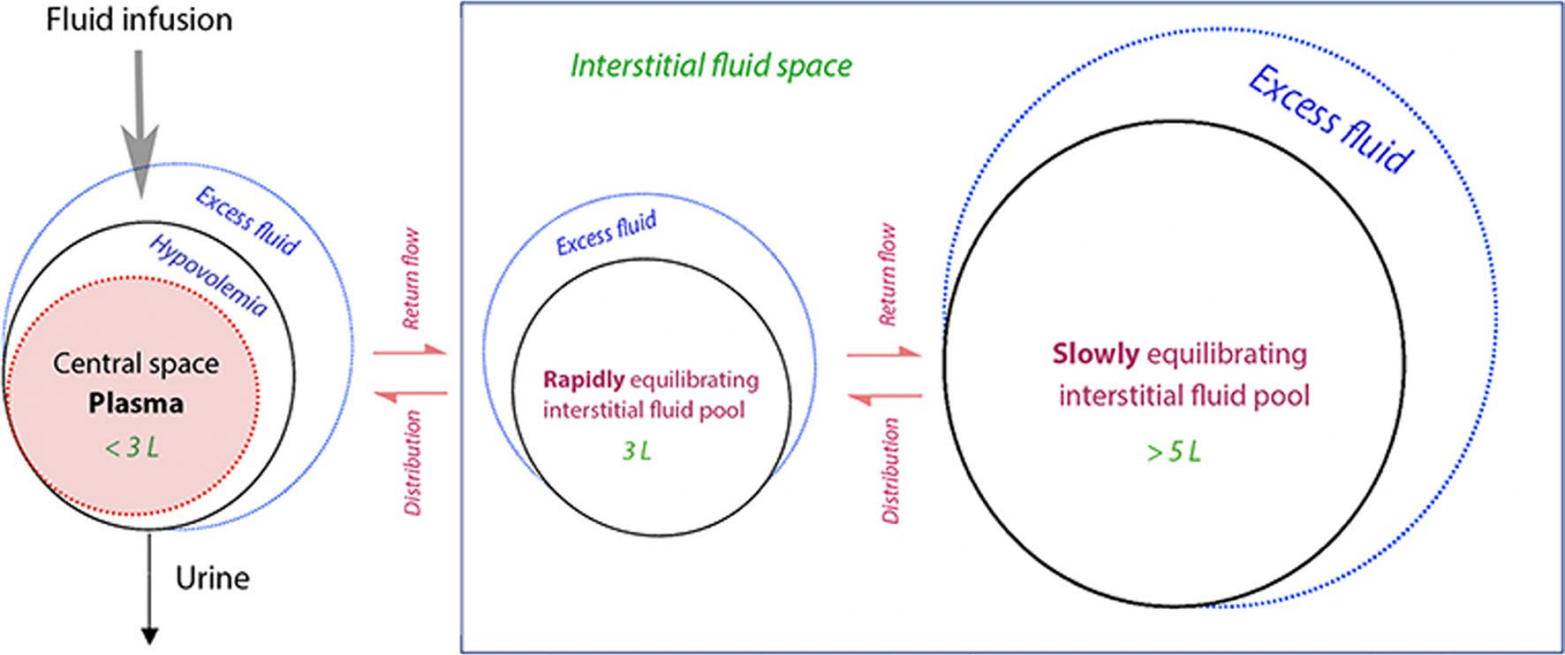
The volume kinetic model used the distinguish between two interstitial fluid pools. Hypovolemia might develop if distribution to the infused fluid to the interstitial pools is prominent

The described microcirculatory and molecular events may be interpreted when having macroscopic analyses in mind. During the past decade, volume kinetic studies have quantified the entrance of isotonic electrolyte fluid to the slowly equilibrating interstitial pool by using the rate constant *k*_b_. In conscious volunteers this parameter attains a value being 10% of rate of entrance to the fast fluid pool, and approximately 25% of the urine flow rate [10]. During general anesthesia *k*_b_ becomes approximately 50% larger, and the flow may even exceed the urinary excretion [3] (Fig. 5).

Pooling of many experiments suggests that fluid leaves the slowly equilibrating fluid pool and returns to the plasma with a half-time of approximately 2 h while the half-time is close to 10 min for the rapidly equilibrating pool (N = 233, unpublished). The lymphatic circuit operates like a “telephone line” and return flow of fluid to the plasma increases within minutes in response to intravenous fluid loading [77].

The half-time for the persistence of fluid in the slow interstitial pool is much longer in inflammatory states. For example, re-analysis of a cohort of patients undergoing surgery for cholecystitis or appendicitis even yielded a negative return flow rate for the slowly equilibrating fluid phase [78]. This result agrees with the bewildering circumstance in burns that a fluid infusion further lowers P_if_ [53]).

Li et al*.* induced sepsis in 25 anesthetized sheep by injecting lipopolysaccharide and puncturing the cecum [29]. One hour later, they studied the volume kinetics of 20 mL/kg of Ringer´s lactate infused over 30 min. Overall, there was virtually no urinary excretion and extremely small or negative flow rates of fluid distribution from the interstitium to the plasma. Refined analysis again showed that the rate constant for the return flow from the slowly equilibrating fluid pool attained a negative value, which suggests a suction effect. This expansion occurred at the expense of the rapidly equilibrating fluid phase, which now amounted to only 1/3 of the plasma volume. These experiments were designed to distinguish the influence of four catecholamines on the distribution of infused Ringer´s, but only dopamine showed a slight ability to “normalize” the clearly pathological fluid distribution.

Preeclampsia during pregnancy is characterized by hypovolemia, hypoalbuminemia, and peripheral edema, and hypertension [79]. A comparison between 8 women with mild to moderately severe preeclampsia and 8 gestational-week matched controls showed that the former had almost complete lack of return flow of infused Ringer´s from the interstitium to the plasma [3]. Refined analysis shows that entrance to the slowly equilibrating interstitial fluid pool occurred 4 times faster in preeclampsia than in healthy pregnant and non-pregnant women, while the size of the rapidly equilibrating pool had shrunk to a negligible volume.

Kwashiorkor is a nutritional disease which shares the pathological fluid distribution with sepsis. Hypoalbuminemia is an obligate finding but not enough to explain the peripheral edema. Recently, Gonzalez et al*.* reported that the plasma concentrations of lumican (an interstitial matrix protein) and metalloproteinase-2 (involved in the breakdown of the interstitial matrix) increase with the degree of edema. By contrast, biomarkers of endothelial integrity (syndecan-1 and hyaluronan) show an inverse relationship with the edema [80]. These findings suggest that molecular events in the interstitium, other than inflammation, account for the pathological fluid distribution.

## Limitations

A warning is often given when pharmacokinetic data is interpreted into physiological terms, which is frequently warranted, due to tissue binding issues and drug metabolism. Volume kinetics provide functional volumes and rates for fluids and have long given reasonable estimates of plasma volumes and descriptions of physiological events. Currently, it is the best available method by which dynamic whole-body events related to fluid infusions can be understood.

Our data suggest that the rapidly equilibrating fluid pool correspond to the interstitial “free fluid phase” and likely to fluid in very close proximity to capillaries, and that the slowly equilibrating pool corresponds to the “gel phase” or interstitial space remote from capillaries or lymphatic. Our data further suggest that the interstitial free fluid phase normally occupies more volume than the 1% suggested by physiological studies, but the volume may also become very small, probably by compression, when the slowly equilibrating fluid phase swells.

## Conclusions

We have reviewed molecular and macroscopic evidence showing that the interstitial space plays an active role in the development of pathological fluid distributions characterized by hypovolemia, hypoalbuminemia, and peripheral edema. An important pathway consists of swelling of the gel phase due to loss of integrin-cellular interactions caused by inflammatory signals. This dramatically reduces the interstitial hydrostatic pressure which applies a suction effect on the plasma and prolongs the time period when distributed fluid resides in the interstitium. Associated actions of nitric oxide involves an inhibition of lymphatic pumping. These events are reflected in macroscopic studies using volume kinetics, which identifies a small interstitial pool of rapidly exchanging fluid and a large pool which equilibrates slowly. General anesthesia modestly increases of the absorption of fluid into the slowly exchanging pool. Inflammatory disorders (cholecystitis and sepsis) greatly prolong the time period when the infused fluid resides in the slowly exchanging fluid pool, and the resulting swelling occurs in part at the expense of the size of the rapidly equilibrating pool.

## Data Availability

All data was from referenced published works.

## Abbreviations

P_if_: Interstitial fluid pressure
P_I_: Interstitial hydrostatic pressure
NO: Nitric oxide
